# Papillary Thyroid Carcinoma Arising in Children and Adolescent Hashimoto's Thyroiditis: Ultrasonographic and Pathologic Findings

**DOI:** 10.1155/2016/2397690

**Published:** 2016-02-10

**Authors:** Sun Hye Jeong, Hyun Sook Hong, Eun Hye Lee, Jeong Ja Kwak

**Affiliations:** ^1^Department of Radiology, Soonchunhyang University Bucheon Hospital, Bucheon 14584, Republic of Korea; ^2^Department of Pathology, Soonchunhyang University Bucheon Hospital, Bucheon 14584, Republic of Korea

## Abstract

*Objectives*. We compared the ultrasonography and pathology features of papillary thyroid carcinoma (PTC) in pediatric and adolescents with Hashimoto's thyroiditis (HT) with those of non-HT patients.* Materials and Methods*. Eleven patients who were surgically confirmed to have pediatric or adolescent PTC from 2006 to 2014 were included in this study. We retrospectively analyzed the preoperative ultrasonography and pathology features of PTC arising in HT and non-HT patients.* Results*. On ultrasonography, thyroid gland was lobulated and enlarged, with many scattered microcalcifications in four of five HT patients. Four of six non-HT patients had suspicious masses with calcifications. The diffuse sclerosing variant of PTC (DSVPTC) was found in three of five HT patients, but none in non-HT patients. Macroscopic or microscopic extrathyroidal extension was evident in all of the HT patients and four of the non-HT patients. Neck lymph node metastases were in all HT patients and five of non-HT patients.* Conclusions*. Three of five PTCs in pediatric and adolescent HT patients were DSVPTC, whereas all PTCs of the non-HT patients were classic type. On ultrasonography, thyroid gland was diffusely enlarged with scattered microcalcifications in four of five HT patients. All five HT cases had aggressive disease, including extrathyroidal extension and cervical lymph node metastases.

## 1. Introduction

Thyroid carcinoma is uncommon in children, constituting 0.5~3% of all pediatric malignancies [1–4]. Papillary thyroid carcinoma (PTC) is the most common type of pediatric and adult thyroid carcinoma [5]. Hashimoto's thyroiditis (HT) is the most common form of diffuse thyroid disease, which is characterized by diffuse lymphocytic infiltration, and affects 1.3% of children and adolescents [5–7]. Some evidence suggests that HT patients are at an increased risk of PTC compared to the general population [8–10]. However, as PTC is rare in pediatric and adolescent HT patients, the carcinoma has been poorly studied. Therefore, we compared the ultrasonography and pathology features of PTC arising in pediatric and adolescent HT, and non-HT patients.

## 2. Materials and Methods

### 2.1. Patients

Institutional Review Board approval was obtained for this retrospective study and the requirement for informed patient consent was waived.

Thirteen patients were diagnosed with pediatric or adolescent PTC in our institution from July 2006 to April 2014. Of these, this study enrolled the 11 patients who underwent surgery. Two patients were excluded: one because, although PTC was diagnosed by fine needle aspiration biopsy (FNAB) in our institution, the patient was subsequently transferred to another hospital, and the other had undergone radiofrequency ablation in another hospital before PTC was diagnosed, which likely affected the characteristics of the mass. We retrospectively analyzed the preoperative ultrasonography and/or laboratory findings. All thyroid cancers were examined by a pathologist and the diagnoses were confirmed after subsequent surgery.

### 2.2. Laboratory Findings

The serum triiodothyronine (T3), thyroxine (T4), free T4, thyroid-stimulating hormone (TSH), anti-microsomal antibody (AMA), anti-TSH-receptor antibody, anti-thyroglobulin antibody (ATA), and thyroglobulin (Tg) levels were recorded.

### 2.3. Thyroid Ultrasonography and Image Analysis

Ultrasonography and Doppler examinations were performed using 5–12 MHz linear array transducers (LAT) (IU22 US or HDI 5000; Philips Healthcare, Bothell, WA); 6–15 MHz LAT (LOGIQ E9; GE Healthcare, Milwaukee, WI); or 5–10 MHz LAT (LOGIQ 700; GE Healthcare).

Two radiologists with 25 and 5 years of experience, who specialized in thyroid imaging, independently reviewed all ultrasonography images while blinded to the clinical information and original interpretations and then consensus was achieved. The criteria for grayscale grading of the ultrasonography data were adapted from those of previous studies [11–13].

### 2.4. Pathology

Ultrasound-guided FNAB was performed with the aid of a 22-gauge needle attached to a 10 mL disposable plastic syringe rinsed in a methanol-water solution (ThinPrep CytoLyt, Hologic, Crawley, West Sussex, UK). Each lesion was aspirated at least twice. Two cytopathologists with 23 and 21 years of experience interpreted the FNAB slides.

Sections were stained with hematoxylin and eosin (H&E) and histologically evaluated by a pathologist (JJK) to diagnose PTC (including the variant type) and to determine if lymphocytic thyroiditis was evident in the thyroid gland overall. Numerous psammoma bodies, squamous metaplasia, and marked sclerosis were the criteria used to diagnose the diffuse sclerosing variant of PTC (DSVPTC) [14]. As Mizukami et al. suggested [15], only cases exhibiting lymphoplasmacytic infiltration with germinative center formation, oxyphilic cell metaplasia (Hürthle), atrophy, and thyroid follicle fibrosis (also called “signs of chronic oxyphilic lymphocytic thyroiditis”) were diagnosed as HT histopathologically, provided that the AMA or ATA status was positive.

The location, size, and histological features of each tumor were noted.

Lymph node status was defined by any pathological evidence of metastasis to the lymph nodes that were removed. Extraglandular involvement was evidenced by tumor infiltration beyond the capsule of the gland, on macroscopic or microscopic examination.

## 3. Results

The clinical, ultrasonography, and pathology findings of the 11 patients are summarized in Table 1. We treated two males and females ranging in age from 6 to 19 years. These included five HT patients (cases 1–5) and six non-HT patients (cases 6–11). Among them, two non-HT patients did not undergo preoperative ultrasonography.

**Figure 1:**
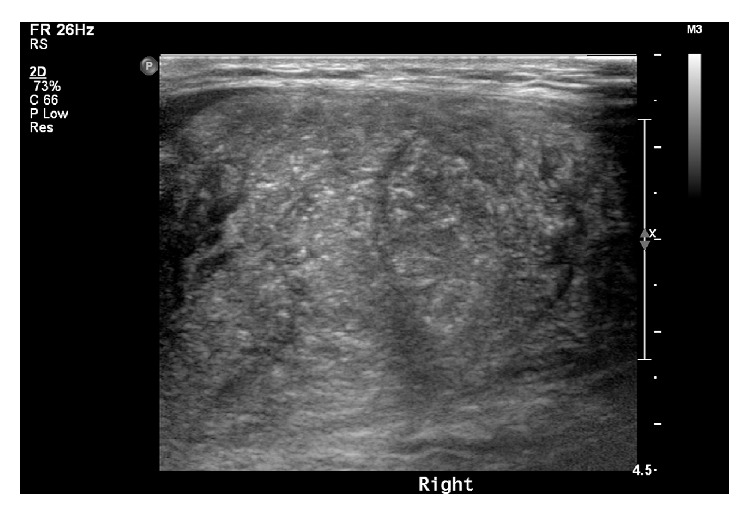
Thyroid ultrasonography of a 13-year-old girl with Hashimoto's thyroiditis. Enlarged, lobulated, diffusely contoured thyroid lobes with many scattered microcalcifications are evident.

**Figure 2:**
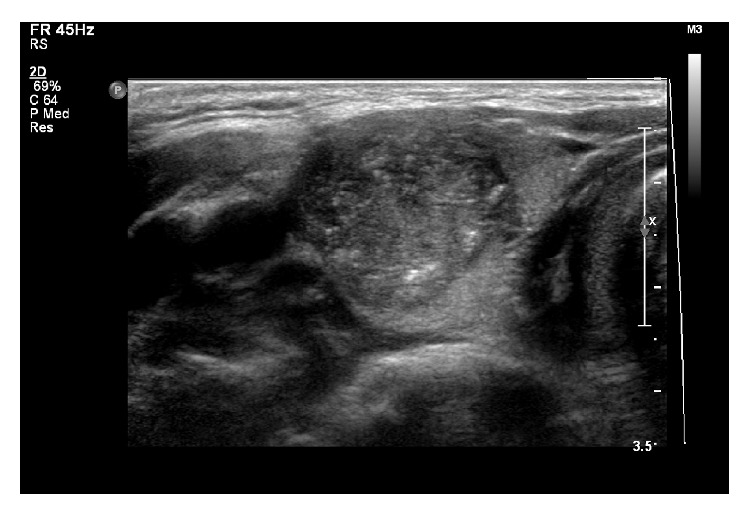
Thyroid ultrasonography of an 18-year-old girl with Hashimoto's thyroiditis. An oval poorly defined hypoechoic solid nodule containing internal microcalcifications which is 2.7 cm in diameter is evident in the right thyroid gland.

In all five HT patients, neck ultrasonography showed that both thyroid lobes were enlarged, lobulated contoured, and with many scattered microcalcifications in four of the patients (cases 1–4) (Figure 1). Case 5 had an oval poorly defined hypoechoic solid nodule containing internal microcalcifications which is 2.7 cm in diameter, in the right thyroid gland (Figure 2). In all five HT patients, the thyroid glands exhibited heterogeneous parenchymal echogenicity and diffusely increased vascularity on color Doppler analysis, indicative of underlying thyroiditis.

All four non-HT patients who underwent preoperative ultrasonography had suspicious masses with inner calcifications in the thyroid.

The ultrasonography-guided FNAB or core biopsy results were PTC (*n* = 6), PTC suspicion (*n* = 3), and an indeterminate follicular cell lesion (*n* = 2).

**Figure 3:**
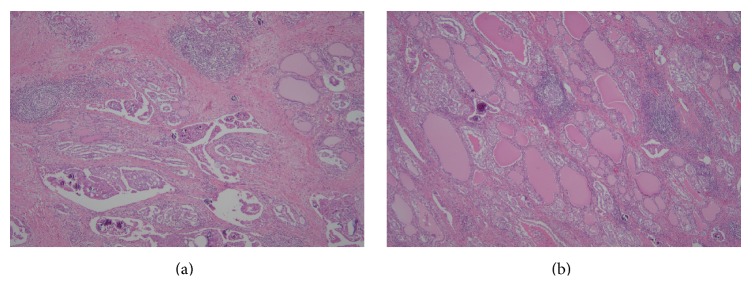
Microscopically, diffusely scattered tumor aggregates, extensive fibrosis, marked lymphocytic infiltration with formation of lymphoid follicles, and abundant psammoma bodies (a) were evident against a background of Hashimoto's thyroiditis (b) (Hematoxylin & Eosin stain ×40).

**Figure 4:**
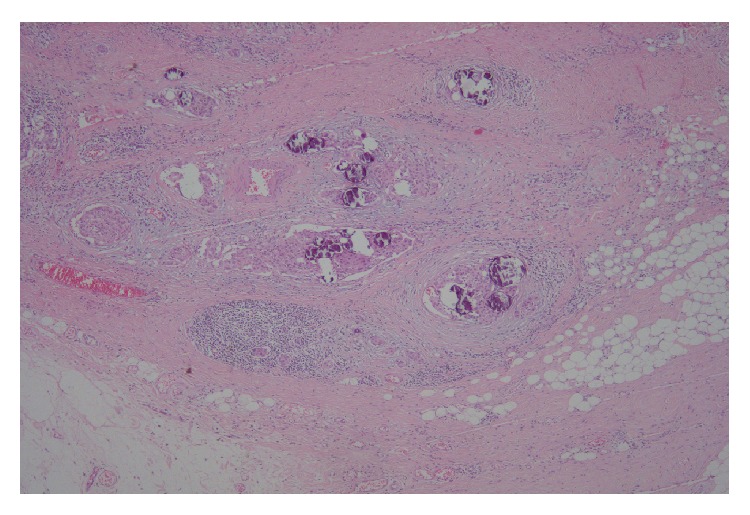
Extrathyroidal extension of papillary thyroid carcinoma was noted (H-E stain ×40).

**Figure 5:**
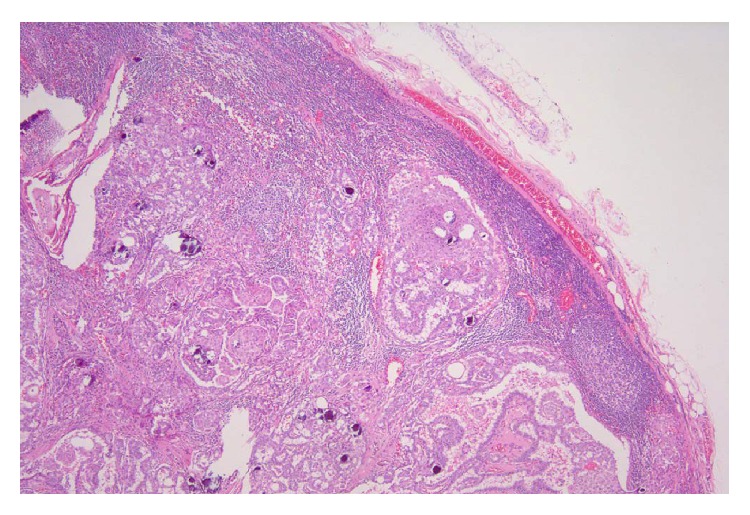
The lymph nodes exhibit metastatic papillary carcinoma of the classical configuration, with characteristic nuclei, and numerous microcalcifications (Hematoxylin & Eosin stain ×40).

All patients underwent subsequent thyroidectomy (total: 10; left-side: 1); the final pathology was PTC in all cases. Tumors were located in both lobes (*n* = 4) or the left (*n* = 3) or right (*n* = 4) lobes. Three DSVPTCs were noted in HT patients (60%) (Figures 3(a) and 3(b)), but not in non-HT patients (0/6, 0%). Pathologically, calcifications were evident in 10 cases (psammomatous: 9; dystrophic: 1). Macroscopic extrathyroidal extensions were evident in four of the HT patients (4/5, 80%) (Figure 4) and three of the non-HT patients (3/6, 50%). Microscopic extrathyroidal extensions were seen in two patients (HT: 1; non-HT: 1). Uni- or bilateral neck lymph node metastases were evident in 10 patients (5/5, 100% of HT patients; 5/6, 83.3% of non-HT patients) (Figure 5). Case 3 had a thymic metastasis.

Of the five HT patients, two had hyperthyroidism, two had subclinical hypothyroidism (an elevated TSH, but a normal free T4 level), and the remaining patient was euthyroid. Three patients were positive for both AMA and ATA antibodies; two of these patients were also positive for anti-TSH-receptor antibody. The remaining two patients were only anti-thyroglobulin antibody-positive. The serum Tg levels were normal in all five HT patients.

Of the six non-HT patients, three were euthyroid, and one each had hyperthyroidism, subclinical hypothyroidism, and hypothyroidism. The serum Tg levels were high in three patients (3/6, 50%).

## 4. Discussion

Since Dailey et al. [16] first described a significant relationship between PTC and HT in 1955, the association has been debated vigorously [17]. One meta-analysis [18] found that pathologically confirmed HT was more often present in PTC cases than in patients with benign thyroid diseases or other carcinomas. In the cited meta-analysis, the mean ages of the PTC patients with HT ranged from 39.5 to 69.0 years [18].

As PTC is rare, there are only a few reports on the ultrasonography and pathology features of tumors in children or adolescents with HT. Koibuchi et al. reported the ultrasonography findings in three pediatric PTC cases with associated HT. In all three cases, irregular hypoechoic nodules with microcalcifications were evident in the thyroid gland [19]. In their study, variant types of PTC were not discussed. In our study, 4 of the 5 cases (80%) had diffusely scattered microcalcifications both ultrasonographically and pathologically. By contrast, all four non-HT patients who underwent preoperative ultrasonography had suspicious masses with inner calcifications in the thyroid gland. We presume that the ultrasonographic differences of PTCs between HT and non-HT patients are explained by variations in PTC type. Three of five HT patients (3/5, 60%) had DSVPTC, which is characterized as ultrasonographically heterogeneous echogenicity with diffusely scattered microcalcifications [20–22]. By contrast, all PTCs of non-HT patients were of the conventional type. The reported incidence of DSVPTC in the general population (mainly adults) is 0.3–5.3% [21, 23, 24]. There is no well-established relationship between HT and DSVPTC. Two reports described 15- and 18-year-old girls with DSVPTC presenting as HT [25, 26]. Those authors did not address an association between PTC and HT; however, they suggested that because the echo structures are similar, the presence of HT may delay a diagnosis of DSVPTC.

Microscopically, DSVPTC is characterized by the following five features: diffuse gland involvement; dense and sclerotic fibrosis; extensive lymphocytic infiltration; numerous psammoma bodies; and frequent squamous metaplasia. Infiltration of the gland by lymphocytes and plasma cells are the most characteristic feature of HT. As abundant lymphocytic infiltration is characteristic of both DSVPTC and HT, we presume that DSVPTC is associated with HT. It is very important to distinguish DSVPTC from classical PTC in HT patients, because the former condition is associated with massive thyroid gland involvement and higher rates of local and distant metastasis at presentation [20, 21]. Therefore, DSVPTC patients should be treated aggressively and followed closely. The American Thyroid Association Guidelines for managing children with autoimmune thyroiditis recommend thyroid ultrasonography if there is palpable thyroid nodule or asymmetry and consideration of FNA for nodules which show suspicious sonographic features or growth over time [27]. On the basis of our study, we also recommend more close ultrasonography follow-up and FNA for suspicious features including diffuse microcalcifications should be considered for pediatric and adolescent HT patients.

Our study has some limitations. We studied only a small number of cases treated in a single institution. Larger cohort studies including adults are required. Additionally, two of six non-HT patients did not undergo preoperative ultrasonography. They were analyzed by contrast enhanced CT instead of ultrasonography.

## 5. Conclusions

Three of five PTCs in pediatric and adolescent HT patients were of the diffuse sclerosing variant, whereas all PTCs of the non-HT patients were classic type. Ultrasonographically, both thyroid lobes were enlarged with diffusely scattered microcalcifications in four of five HT patients. The cancers showed aggressive features, including extrathyroidal extensions and cervical lymph node metastases, compared to the PTCs of non-HT patients.

## Figures and Tables

**Table 1:** Clinical and ultrasonographic, pathologic features of patients with papillary thyroid carcinoma.

Case	Age/sex	Site	US findings	FNA or core biopsy	Op	Op pathology	Diffuse calcifications on pathology	Diffuse sclerosing variant	Lymphocytic thyroiditis	Extrathyroidal extension	Type of calcifications on pathology	Rt neck nodes	Lt neck nodes
1	12/F	Both	Diffuse calcifications	Suspicious for PTC (FNA)	TT with both RND	PTC	Yes	No	Yes	Yes	Psammomatous	II–IV, VI	II–IV, VI

2	12/F	Lt	Diffuse calcifications	Follicular cell lesion, indeterminate (FNA)	TT with left RND	PTC	Yes	Yes	Yes	Yes	Psammomatous	No	II, III, VI

3	13/F	Both	Diffuse calcifications	PTC (core-biopsy)	TT with both RND	PTC	Yes	Yes	Yes	Yes (with thymic metastasis)	Psammomatous	II–IV, VI	II–IV, VI

4	18/F	Both	Diffuse calcifications	PTC (FNA)	TT with both RND	PTC	Yes	Yes	Yes	Yes (Microscopic extension)	Psammomatous	II, III	II–IV, VI, delphian node

5	18/F	Rt	Mass with internal calcifications	PTC (FNA)	TT with right RND	PTC	Yes	No	Yes	Yes	Psammomatous	II–IV, VI	N/A

6	6/F	Both	Oval well defined hypoechoic mass with calcifications	Suspicious for PTC (FNA)	TT with both RND	PTC	Yes	No	No	Yes	Psammomatous	II–IV, VI	II–IV, VI

7	18/M	Lt	Oval ill-defined isoechoic mass with calcifications	Suspicious for PTC (FNA)	TT with left RND	PTC	Yes	No	No	Yes	Psammomatous	No	II–IV

8	19/F	Rt	Taller than wide hypoechoic mass with calcifications	PTC (FNA)	TT with right RND	PTC	No	No	No	No	No	V, VI	No

9	17/M	Lt	N/A	Follicular cell lesion (FNA)	Lt thyroidectomy	PTC	No	No	No	No	Psammomatous	No	No

10	18/F	Rt	N/A	PTC (FNA)	TT with right RND	PTC	No	No	Yes	Yes	Dystrophic	II–VI	No

11	19/F	Rt	Mass with calcifications	PTC (FNA)	TT with right RND	PTC	No	No	Yes	Yes (Microscopic extension)	Psammomatous	II–IV, VI	No

US: ultrasonography; Op: operation or type of surgery; Rt: right; Lt: left; FNA: fine needle aspiration; PTC: papillary thyroid carcinoma; TT: total thyroidectomy; RND: radical neck dissection; N/A: not applicable.
